# Genome-Wide Analysis of MicroRNAs in Relation to Pupariation in Oriental Fruit Fly

**DOI:** 10.3389/fphys.2019.00301

**Published:** 2019-03-22

**Authors:** Qiang Zhang, Wei Dou, Deng Pan, Er-Hu Chen, Jin-Zhi Niu, Guy Smagghe, Jin-Jun Wang

**Affiliations:** ^1^Key Laboratory of Entomology and Pest Control Engineering, College of Plant Protection, Southwest University, Chongqing, China; ^2^Academy of Agricultural Sciences, Southwest University, Chongqing, China; ^3^International China-Belgium Joint Laboratory on Sustainable Crop Pest Control Between Southwest University in China and Ghent University in Belgium, Chongqing, China; ^4^Department of Plants and Crops, Ghent University, Ghent, Belgium

**Keywords:** miRNAs, *Bactrocera dorsalis*, pupariation, metamorphosis, gene expression

## Abstract

Insect metamorphosis is a complex process involving drastic morphological and physiological changes. microRNAs (miRNAs) are a class of endogenous small non-coding RNAs that play key roles in regulating various biological processes, including metamorphosis, by post-transcriptional repression of mRNAs. The oriental fruit fly, *Bactrocera dorsalis*, is one of the most destructive insect pests in many Asian countries and the Pacific Islands. The regulatory role of miRNAs in *B. dorsalis* metamorphosis is unclear. To better understand the molecular regulatory mechanisms of miRNAs in pupariation, Illumina sequencing of the wandering stage (WS), the late WS and the white puparium stage of *B. dorsalis* were performed. Two hundred forty-nine miRNAs, including 184 known miRNAs and 65 novel miRNAs, were obtained. Among these miRNAs, 19 miRNAs were differentially expressed in pupariation, and eight miRNAs showed relative high expression levels (>50 TPM), of which five differentially expressed miRNAs (DEMs) had target differentially expressed genes (DEGs) predicted by the expected miRNA–mRNA negative regulation pattern using the Illumina HiSeq data. Four sets of DEMs and their predicted target DEGs were confirmed by qPCR. Of the four miRNAs, two miRNAs were down-regulated: miR-981, which may target *pdpc*, and Bdo-novel-mir-55, which potentially regulates *spsX1, psB/C*, and *chit3*. The other two miRNAs were up-regulated: let-7a-3p, which possibly controls *lap*, and Bdo-novel-mir-24, which may regulate *ipc* and *sp1/2*. This study provides a useful resource to elucidate the regulatory role of miRNAs and understand the molecular mechanisms of metamorphosis.

## Introduction

Metamorphosis is an important process, because pupation failure means death, and also a complex process involving extensive morphological, biochemical, and cellular changes. During larval–pupal metamorphosis, the soft cuticle of a wandering larva is transformed into a hard puparium, which involves immobilization, cuticular shrinkage, anterior retraction, longitudinal body contraction, and tanning of the cuticle (Jan and Fraenkel, 2010). In holometabolous insects, a short pulse of ecdysone peak occurs in larval–pupal metamorphosis (Dubrovsky, 2005), and the obsolete larval tissues or organs (including the integument, midgut, and fat body) undergo a remodeling process during larval–pupal metamorphosis, involving programmed cell death and histolysis of the tissues or organs to recycle intracellular components for formation of the pupal-adult organs (Yang et al., 2017; Guo S. Y. et al., 2018). The regulatory role of hormones is relatively clear during insect metamorphosis. For example, juvenile hormone (JH) and 20-hydroxyecdysone (20E) jointly regulate larval–pupal development. While both hormones are present at the larval stages, they have opposing roles in development. The role of JH is to maintain insects at the larval stage, whereas 20E induces metamorphosis. At the end of the last larval stage, the 20E titer increases while the JH titer decreases, and then the metamorphosis occurs (Liu S. et al., 2017; Zhang T. et al., 2018). Additionally, as chitin is a crucial component of the cuticle, insect metamorphosis is largely dependent on chitin degradation and synthesis, which are strictly coordinate and occur almost simultaneously during larval–pupal development (Yao et al., 2010). Thus, a series of chitin biosynthesis enzymes (such as chitin synthase) and catabolic pathway enzymes (such as chitinase) control the process of metamorphosis by regulating chitin.

However, many details of the regulatory networks involved in pupariation remain undefined. For example, it is not known how regulatory non-coding RNAs (such as long non-coding RNAs, small interfering RNAs, piwi-interacting RNAs, circular RNAs, or miRNAs) regulate pupariation. In these regulatory non-coding RNAs, miRNAs are our focus in the present study. miRNAs are 18–25 nucleotide (nt) RNAs that post-transcriptionally regulate gene expression through interactions with their target transcripts (Reynolds et al., 2017). Changes in miRNA profiles have been observed during immune responses (Garbuzov and Tatar, 2014; Yin et al., 2018), reproduction (Tariq et al., 2016; Zhang et al., 2016) and insecticide resistance (Etebari et al., 2018; Zhang Y. et al., 2018). In addition, they have been implicated as regulators of metamorphosis, involving in the JH, 20E and chitin pathways. miR-278 and let-7 regulated the early JH-response gene, *Kr-h1*, in *Locusta migratoria* (Song et al., 2018). miR-14 controlled 20E signaling gene, *E75*, and the ecdysone receptor, *EcR-B*, in *Bombyx mori* (Liu Z. et al., 2017). miR-173 directly targeted 20E signaling gene, Ftz-F1, and miR-2703 regulated *chitin synthasegene A* in *Nilaparvata lugens* (Li T. et al., 2017; Chen J. et al., 2018). miR-281-3p regulated EcR of *Plutella xylostella* (Wang et al., 2018).

The oriental fruit fly, *Bactrocera dorsalis* (Hendel) (Diptera: Tephritidae), is one of the most destructive pests in Southeast Asia and several Pacific Islands. They cause damage to over 250 different fruits and vegetables, including citrus, guavas, carambolas, mangos, peaches, and chili peppers (Jin et al., 2011; Wang et al., 2014). *B. dorsalis* is highly polyphagous and invasive, and so many countries have imposed strict quarantine restrictions (Shen et al., 2011; Khan and Akram, 2018).

As a dipteran insect, *B. dorsalis* is a typical holometabolous insect that undergoes larval–pupal metamorphosis. Additionally, several studies have shown that miRNAs are involved in *B. dorsalis* development and reproduction (Huang et al., 2014; Peng et al., 2016, 2017). Considering the diverse roles of miRNAs, some miRNAs in the regulation of gene expression during metamorphosis probably exist in *B. dorsalis*. Therefore, we constructed and sequenced small RNA libraries from *B. dorsalis* at three developmental stages, including the wandering stage (WS), the late wandering stage (LWS, the body contraction just before pupariation), and the white puparium stage (WPS). We identified known and novel miRNAs, based on the result of Illumina HiSeq, and analyzed miRNAs expression profile during the different developmental stages. Additionally, to confirm the five differentially expressed miRNAs (DEMs) and differentially expressed genes (DEGs) identified by bioinformatics analysis, the qPCR was performed. The current study provides a useful resource to elucidate the regulatory role of miRNAs and understand the molecular mechanisms of metamorphosis.

## Materials and Methods

### Sample Preparation and RNA Extraction

The oriental fruit fly was reared as described previously (Huang et al., 2014). Briefly, *B. dorsalis* were reared at 27 ± 1°C and 70 ± 5% relative humidity with a photoperiod regime of 14:10 h light:darkness. The larvae were reared on an artificial diet containing corn, yeast powder, wheat flour, and sucrose. Three replicates were performed for each of the three developmental stages (WS, LWS, and WPS), and each replicate contained eight flies. Total RNA was extracted using the RNeasy plus Micro Kit (Qiagen, Hilden, Germany) following the manufacturer’s instructions. In brief, the absorbance at 260 nm was used to quantify the RNA using a NanoVue UV-Vis spectrophotometer (GE Healthcare Bio-Science, Uppsala, Sweden). The absorbance ratios of OD260/280 and OD260/230 were used to test the purity of all RNA samples. The integrity of the RNA was assessed by 1% agarose gel electrophoresis.

### cDNA Library Construction and HiSeq Sequencing

A total amount of 1.5 μg RNA per sample was used as input material and made up to a volume of 6 μL with nuclease-free water for the RNA sample preparations. Sequencing libraries were generated using NEB Next Ultra small RNA Sample Library Prep Kit for Illumina (San Diego, CA, United States) following manufacturer’s recommendations, and barcodes were added to the adaptor sequences for each sample. Briefly, because of the phosphate group at the 5′ end of the small RNA and the hydroxyl group at the 3′ end, T4 RNA Ligase 1 and T4 RNA Ligase 2 (truncated), respectively, were used to ligate the adaptors at the ends of the small RNA fragments. cDNA was synthesized by reverse transcription and then amplified by PCR, and the target fragments were screened using glue separation technology. The recovered fragments were cut in the same way as the small RNA library (sRNA). After completion of library construction, the concentration of the library was determined using a Qubit 2, and then the library was diluted to 1 ng/μL. Subsequently, to guarantee the quality of the library, a 2100 Bioanalyzer (Agilent, Santa Clara, CA, United States) was used to detect the insert size, and qPCR was used to accurately quantify the effective concentration of the library.

The clustering of the barcoded samples was performed on a cBot Cluster Generation System (Illumina) using the TruSeq PE Cluster Kit v4-cBot-HS (Illumina) according to the manufacturer’s instructions. After cluster generation, the library preparations were sequenced on an Illumina HiSeq 2500 platform and paired-end reads were generated.

### Analysis of HiSeq Data

Raw sequence reads (raw data) were acquired by the Illumina HiSeq X-ten platform. Clean reads (clean data) were obtained by removing reads containing ‘N’ (an unrecognized base) at 10% or higher, reads with no adaptor sequence at the 3′ end, reads with a sequence shorter than 18 nt or more than 30 nt, low quality reads, and reads with truncated adaptor sequences at the 3′ end. The Q20, Q30, GC content and sequence duplication level of the clean data were calculated. Subsequently, using Bowtie software, the clean reads were compared with the Silva, GtRNAdb, Rfam and Repbase databases, filtering out ribosomal RNA (rRNA), small cytoplasmic RNA (scRNA), transfer RNA (tRNA), small nuclear RNA (snRNA), small nucleolar RNA (snoRNA) and repeats, to give unannotated reads. The unannotated reads were prepared for further analysis by mapping them with the *B. dorsalis* genome (NCBI Assembly: ASM78921v2). Based on the biological characteristics of miRNA and the mapping to the reference genome sequence, miRDeep2 (Friedlander et al., 2012) software was used to make the identification of known miRNAs with the hairpin precursors in miRBase21^1^. Meanwhile, based on possible precursor sequences obtained by mapping reads to the genomic location, the distribution of reads on the precursor sequence and the precursor structure energy information (RNAfold), a Bayesian model was used to identify novel miRNAs. The miRNA expression levels were further quantified by the TPM (Transcripts Per Kilobase of exon model per Million mapped reads) method (Fahlgren et al., 2007). In this study, differential expression analysis of two developmental stages (WS vs. LWS and LWS vs. WPS) was performed using the DESeq R package (1.10.1). DESeq provides statistical routines for determining differential expression in digital miRNA expression data using a model based on a negative binomial distribution. miRNAs which normalized expression of 0 were modified to 0.01, and the normalized data were used to calculate a log_2_-ratio plot. The fold-change (log_2_ > 1 or log_2_ < -1) of miRNAs was considered to be differentially expressed.

### Target Gene Prediction and Annotation

Prediction of the target genes of the miRNAs was conducted using the miRanda and RNAhybrid software packages. Gene function was annotated based on the following databases: NCBI non-redundant protein sequences (Nr), Protein family (Pfam), Clusters of Orthologous Groups of proteins (COG), a manually annotated and reviewed protein sequence database (Swiss-Prot), the evolutionary genealogy of genes: Non-supervised Orthologous Groups (eggNOG), Kyoto Encyclopedia of Genes and Genomes (KEGG Ortholog database), and Gene Ontology (GO).

Gene Ontology analysis of DEGs was implemented by the GOseq R packages based Wallenius non-central hyper-geometric distribution. For the COG annotation, the DEGs between two developmental stages (WS vs. LWS and LWS vs. WPS) were translated into amino acid sequences by using the virtual ribosome package. We used the batch web CD search tool^2^ to assign COG groups. For the KEGG annotation, KOBAS software (Mao et al., 2005) was used to test the statistical enrichment of differential expression genes in KEGG pathways.

### Quantitative Real-Time PCR (qPCR)

In this study, qPCR was carried out to confirm the miRNAs and their predicted target genes from the Illumina sequencing results. Total RNA was extracted from the three developmental stages of WS, LWS, and WPS using TRIzol reagent.

For mRNA qPCR, reverse transcription was carried out using PrimeScript RT reagent Kit (Takara, Dalian, China) according to the manufacturer’s protocols. All the primers (Supplementary Table S1) were designed by NCBI Primer Blast^3^. A standard curve for each primer pair was carried out with serial dilutions of cDNA (1, 1/4, 1/16, 1/64, 1/256) to determine the amplification efficiency and cycle threshold (Ct) value. The qPCR was performed on a BIO-RAD Real-Time PCR System (Bio-Rad, Hercules, CA, United States) using the Novostar-SYBR Supermix (Novoprotein, Shanghai, China) reagent, with an initial denaturation at 95°C for 2 min, followed by 40 cycles of 95°C for 15 s and 60°C for 1 min. At the end of the procedure, a melting curve analysis was recorded from 60 to 95°C to ensure the specificity of each pair of primers. The amplification was conducted in a 20 μL reaction volume consisting of 10 μL of SYBR Supermix, 1 μL each of forward and reverse primers (10 μM), 7 μL of nuclease-free water, and 1 μL of cDNA. The relative expression levels were normalized using *α-tubulin* (GenBank Accession No. GU269902) and *rps3* (XM_011212815) as internal references, as described previously (Shen et al., 2010; Wei et al., 2015).

For miRNA qPCR, the reverse transcription reagent used was the miRNA cDNA Synthesis Kit (CWBIO, Beijing, China) according to the manufacturer’s protocols. All the primers (Supplementary Table S1) were designed using the miRNA qPCR Assay Kit (CWBIO) protocol. A standard curve for each primer pair (only the forward primer was designed) was carried out with serial dilutions of cDNA (1/1, 1/2, 1/4, 1/8, 1/16) to determine the amplification efficiency and cycle threshold (Ct) value. The cycling conditions were an initial denaturation at 95°C for 10 min, followed by 45 cycles of 95°C for 15 s and 60°C for 1 min. Melting curve analysis was performed in the same way as above. The amplification was conducted in a 20 μL reaction volume consisting of 10 μL of miRNA qPCR mixture, 0.4 μL of forward and reverse primers (10 μM), 8.2 μL of nuclease-free water, and 1 μL of cDNA. The expression of *U6* was used to normalize the expression of miRNA.

### Statistical Analysis

qPCR experiments were performed in four biological replicates, and qBase software (Hellemans et al., 2007) was used to determine the changes in miRNA and target genes during larval–pupal metamorphosis. The qPCR and the corresponding Illumina sequencing results were analyzed with one-way analysis of variance (ANOVA), and the means were separated with Tukey’s HSD *post hoc* test (*P* < 0.05) using SPSS 23.0 software (SPSS, Chicago, IL, United States).

## Results

### Profile of sRNA Libraries

Three sRNA libraries were constructed from the whole body at three developmental stages (WS, LWS, and WPS) to understand the miRNA regulation involved in larval metamorphosis. The samples from each stage were sequenced in triplicate, and there was more than 11.87 M clean data for each sample after strict quality control (Supplementary Table S2). Approximately 174 million clean reads were found, including 63,799,603, 60,700,881, and 49,530,993 reads in the WS, LWS, and WPS stages, respectively. Cleaning up the Rfam reads (rRNA, scRNA, snRNA, snoRNA, tRNA) and removing repeats, the unannotated reads (41,982,118, 33,973,104, and 26,569,248 reads in the WS, LWS, and WPS stages, respectively) were obtained (Table 1) and mapped onto the *B. dorsalis* genome for further analysis (Supplementary Table S3). The length distribution of the clean data for all three developmental stages mostly showed one main peak at 22 nt, which is the typical length of mature miRNAs, and another smaller peak at 26–27 nt, corresponding to piRNA-like sequences (the role of these putative piRNA sequences as regulators of pupariation was excluded from further analysis).

**Table 1:** Distribution of clean reads for different RNA categories in the libraries.

	WS		LWS		WPS	
Types	Reads	%	Reads	%	Reads	%
rRNA	20205691	31.67	24174075	39.82	21462809	43.33
scRNA	0	0.00	0	0.00	0	0.00
snRNA	7	0.00	11	0.00	18	0.00
snoRNA	3311	0.01	1330	0.00	1856	0.00
tRNA	1576796	2.47	2519152	4.15	1466541	2.96
Repbase	31680	0.05	33209	0.05	30521	0.06
Unannotated	41982118	65.80	33973104	55.97	26569248	53.64
Total	63799603	100	60700881	100.00	49530993	100.00

*rRNA, ribosome RNA; scRNA, small cytoplasmic RNA; snRNA, small nuclear RNA; snoRNA, small nucleolar RNA; tRNA, transfer RNA; Repbase, the repeat sequences.*

### Identification of Known and Novel miRNAs

**FIGURE 1:**
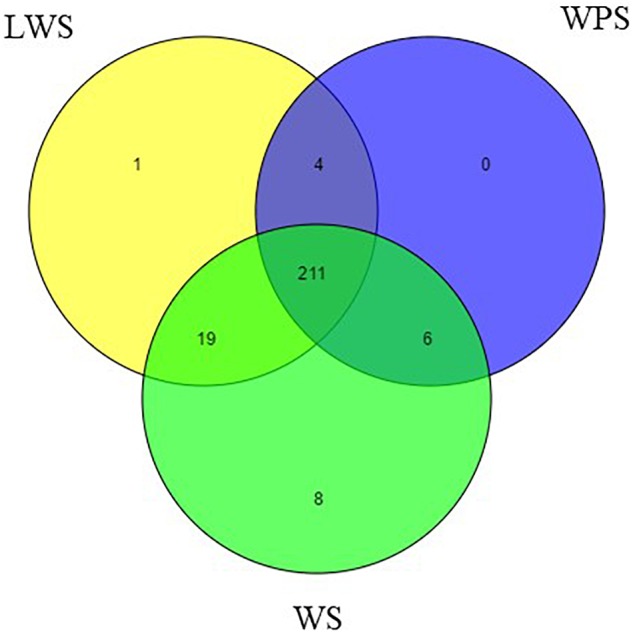
Venn diagram representing the distribution of miRNA in the WS, LWS, and WPS stages of *Bactrocera dorsalis*.

By filtering the data set, the unannotated reads aligned to the *B. dorsalis* reference genome were analyzed. The name of the miRNA with the most reads was used to represent that miRNA and other variants (Peng et al., 2016). Two hundred forty-nine mature miRNAs were obtained, including 184 known miRNAs and 65 novel miRNAs (Supplementary Table S4). Specifically, the samples from WS (244 miNRAs), LWS (235 miRNAs) and WPS (221 miRNAs) yielded 179, 174, and 160 known miRNAs and 65, 61, and 61 novel miRNAs, respectively (Figure 1). The known and novel miRNAs among the three libraries were compared to search for any overlaps, and 152 known and 59 novel miRNAs were observed in all three libraries (Supplementary Table S4).

### Differential Expression of miRNA

**FIGURE 2:**
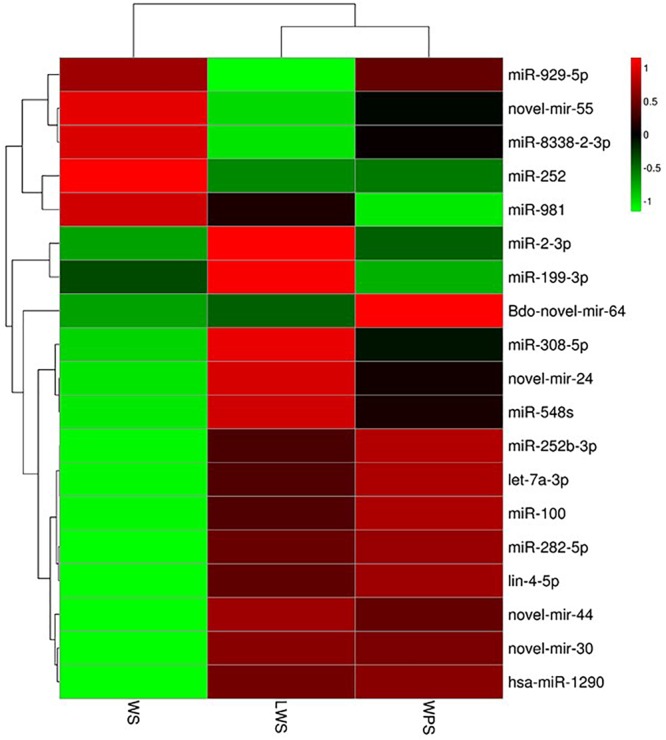
Cluster heat map showing the differential expression of *B. dorsalis* miRNAs over the three stages. (Parameter setting: data adopt the logarithm: log_10_ and normalization; clustering by row and column).

To study the differential expression of miRNAs in the three developmental stages (WS, LWS, and WPS) of *B. dorsalis*, pairwise comparison of the DEMs was conducted, and 89 miRNAs (42 up-regulated and 47 down-regulated, WS vs. LWS) and 81 miRNAs (39 up-regulated and 42 down-regulated, LWS vs. WPS) were differentially expressed (Supplementary Table S5). By filtering the low expression miRNAs (the expression level of any one stage is less than five TPM in the three stages), a total of 19 DEMs was obtained in the current study. Three DEMs (let-7a-3p, miR-2-3p, and Bdo-novel-mir-55) shared between both two comparisons and 16 DEMs were specific (14 and 2 DEMs in WS vs. LWS and LWS vs. WPS, respectively) (Supplementary Table S6). A cluster heat map was subsequently adopted to show the differential expression of *B. dorsalis* miRNAs over the three stages (Figure 2). Eight DEMs showed relative high expression levels (>50 TPM in any comparison stage), where three DEMs (let-7a-3p, miR-2-3p, and Bdo-novel-mir-55) were shared between both two comparisons, miR-282-5p, miR-981, Bdo-novel-mir-24 and Bdo-novel-mir-44 were specific to WS vs. LWS, and miR-100 was unique to LWS vs. WPS.

### Analysis of Differentially Expressed miRNAs and Potential Target mRNAs

According to the sequence information of the known and newly predicted miRNAs, miRNA target gene prediction was conducted. There were 12 (9 up-regulated and 3 down-regulated) and 4 up-regulated DEMs giving 132 and 25 predicted target genes in WS vs. LWS and LWS vs. WPS, respectively (Supplementary Table S7). These were analyzed in combination with the expression data of the differentially expressed target genes; the expression data is from Chen E. H. et al. (2018). There were 10 (7 up-regulated and 3 down-regulated) DEMs associated with 29 predicted DEGs in WS vs. LWS but no one in LWS vs. WPS (Supplementary Table S8). Taking into account the expected pattern of negative regulation between miRNA and mRNA, this resulted in 8 (6 up-regulated and 2 down-regulated) miRNAs associated with 18 predicted DEGs in WS vs. LWS (Supplementary Table S9). Among these DEMs with the predicted DEGs, there were five miRNAs with high expression levels, including three up-regulated miRNAs, let-7a-3p, Bdo-novel-mir-24 and Bdo-novel-mir-44, and two down-regulated miRNA, miR-981 and Bdo-novel-mir-55 that were in WS vs. LWS. Meanwhile, the five miRNAs were significantly different in their expression with *P*-value < 0.05.

### GO, COG, and KEGG Analysis of the Target Genes of the Differentially Expressed miRNAs

To explore the potential function of the DEMs during pupariation, seven databases were used to identify functional modules of the predicted target genes. These databases were GO, KEGG, Nr, COG, Pfam, Swiss-Prot, and eggNOG. There were 132 and 25 predicted target genes in WS vs. LWS and LWS vs. WPS, respectively, that were successfully annotated (Supplementary Table S7). GO, COG, and KEGG analysis was then performed on the predicted DEGs. Because there was no predicted DEG in LWS vs. WPS, the above analysis was only conducted in WS vs. LWS. For the GO classification, the predicted DEGs of the DEMs were divided into three ontologies: biological process, cellular component, and molecular function, including 44 annotations for each comparison. The most enriched functions included ‘Single-organism process,’ ‘Cellular process,’ ‘Metabolic process,’ ‘Binding,’ and ‘Cell’ (Supplementary Figure S1). Furthermore, COG functional classification was performed to compare the enriched functional categories of the predicted DEGs among the three stages using the rpstBlastn program. When mapping the predicted DEGs into the COG database, 11 genes were annotated (Supplementary Table S7). The annotations of the predicted DEGs were mainly focused on ‘general function prediction only,’ followed by ‘Carbohydrate transport and metabolism,’ ‘Amino acid transport and metabolism,’ ‘Replication, recombination and repair,’ and ‘Signal transduction mechanisms’ (Supplementary Figure S2). Additionally, KEGG analysis of the predicted DEGs was used to increase our understanding of the biological functions of the DEMs in metabolism and development of during *B. dorsalis* pupariation. The predicted DEGs were mainly enriched in ‘ECM-receptor interaction,’ followed by ‘Amino sugar and nucleotide sugar metabolism,’ ‘Drug metabolism – cytochrome P450,’ and ‘Glycine, serine, and threonine metabolism’ (Supplementary Figure S3). Furthermore, the Nr annotation of the DEGs of the five highly expressed DEMs included PP2C-like domain-containing protein CG9801 (*pdpc*), corresponding to miR-981; serine proteinase stubble isoform X1 (*spsX1*), protein skeletor, isoforms B/C (*psB/C*), and probable chitinase 3 (*chit3*), all corresponding to Bdo-novel-mir-55; lysosomal aspartic protease-like (*lap*), corresponding to let-7a-3p; collagen alpha-1(IV) chain (*cac*), corresponding to Bdo-novel-mir-44; and putative inorganic phosphate cotransporter (*ipc*) and serine proteases 1/2-like (*sp1/2*), both corresponding to Bdo-novel-mir-24 (Table 2).

**Table 2:** Predicted targets of five high differential expression miRNAs in pupariation of *Bactrocera dorsalis*.

miRNA	miRNA sequences	Number of target genes	*P*-value	log_2_ FC	Predicted target genes
Bdo-novel-mir-24	CGGUCCGUCAUA UGGCAAUCUAAC	15	0.0252	1.1064	*LOC105221956, LOC105222217, LOC105230394, LOC105232269, transcription initiation factor TFIID subunit 7, protein dachsous, neurogenic protein mastermind, putative inorganic phosphate cotransporter, protein phosphatase 1B isoform X1, lysyl oxidase homolog 2A, eukaryotic translation initiation factor 2-alpha kinase 4, voltage-dependent calcium channel subunit alpha-2/delta-3, laminin subunit alpha, serine proteases 1/2-like, protein unc-80 homolog*
Bdo-novel-mir-55	CAUCUGGUAGA CUUUUGGUCCGGC	20	0.0098	–3.0224	*LOC105224189, LOC105233383, cleavage stimulation factor subunit 2, alpha-tubulin N-acetyltransferase 1-like, importin subunit beta, protein Skeletor, isoforms B/C, collagen alpha-1(IV) chain, serine protease gd-like, alpha-mannosidase 2, serine/threonine-protein kinase AtPK2/AtPK19, nuclear pore complex protein Nup160 homolog, serine proteinase stubble isoform X1, mitochondrial ribonuclease P protein 1, protein toll-like, UDP-glucuronosyltransferase 1-8-like, probable chitinase 3, putative inositol monophosphatase 3, GTPase-activating protein CdGAPr, collagen alpha-1(V) chain-like, sodium-dependent nutrient amino acid transporter 1*
Bdo-novel-mir-44	ACCAACGACC AUACCACGCUGA	6	0.0491	1.3237	*Collagen alpha-1(IV) chain, glycoprotein-N-acetylgalactosamine 3-beta-galactosyltransferase 1, transmembrane channel-like protein 7, dyslexia-associated protein KIAA0319-like protein, lysosomal aspartic protease-like, protein krueppel-like*
let-7a-3p	CUAUACAACG UGCUAGCUUUCU	1	1.02E-08	3.3517	*Lysosomal aspartic protease-like*
miR-981	UUCGUUGUCGAC GAAACCUGCA	3	0.0490	–1.2101	*LOC105226582, PP2C-like domain-containing protein CG9801, cytochrome P450 4g1-like*

*log_2_FC, log_2_ fold change.*

### Analysis of Selected miRNA–mRNA Expression Patterns

**FIGURE 3:**
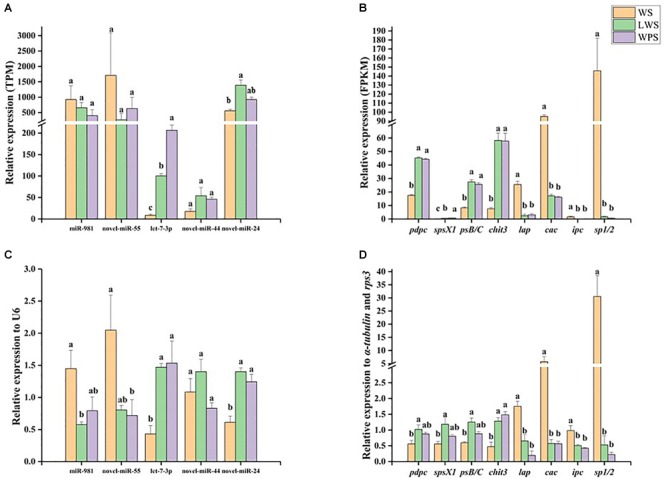
The expression patterns of differentially expressed miRNAs and their predicted differentially expressed mRNA targets were analyzed by quantitative real-time PCR (qPCR) in wandering stage (WS), late wandering stage (LWS, the body contraction just before pupariation) and white puparium stage (WPS) of *Bactrocera dorsalis*. The results of Illumina sequencing also showed here. They included **(A)** relative expression of five miRNAs in Illumina sequencing, **(B)** relative expression of eight targets in Illumina sequencing, **(C)** relative expression of five miRNAs in qPCR, **(D)** relative expression of eight targets in qPCR. The qPCR and Illumina sequencing results were analyzed with one-way analysis of variance (ANOVA), and the means were separated with Tukey’s HSD *post hoc* test (*P* < 0.05). Error bars show the SE. Different letters indicate statistically significant difference among the three stages (*P* < 0.05).

qPCR was used to analyze the miRNA–mRNA expression patterns and validate the Illumina sequencing results. Five DEMs showed relative high expression levels (>50 TPM) with their eight predicted DEGs, in accordance with the expected miRNA–mRNA negative regulation pattern. The results showed that miR-981 and Bdo-novel-miR-55 were significantly down-regulated upon pupariation and that their predicted target genes *pdpc, spsX1, psB/C*, and *chit3* were significantly up-regulated during pupariation. A significant increase in the expression of let-7a-3p and Bdo-novel-mir-24 was observed upon pupariation, and the predicted target genes of those miRNAs, *lap, ipc*, and *sp1/2*, were significantly down-regulated during pupariation. It was observed that Bdo-novel-mir-44 was not significantly changed upon pupariation while its predicted target gene *cac* was significantly down-regulated during this progress (Figure 3). The qPCR results were consistent with the Illumina sequencing results. Furthermore, the miRNA and mRNA expression levels were almost unanimous in the LWS and WPS stages between the qPCR and Illumina sequencing results.

## Discussion

The characteristic role of miRNAs is the post-transcriptional regulation of gene expression that occurs following duplex formation between a transcript and a complementary miRNA, causing subsequent degradation of target mRNAs or inhibition of translational machinery (Bartel, 2009; Belles, 2017). Previous studies have reported insect miRNAs participating in metamorphosis by regulating genes such as the gene encoding the JH-regulated serine protease JHA15 (Lucas et al., 2015), the ecdysone signaling pathway *E75* gene (Peng et al., 2017), and chitin synthase (Li T. et al., 2017). In the present study, we obtained miRNAs at the three stages of metamorphosis development and detected five highly expressed miRNAs and their eight potential target genes, which may participate in the regulation of metamorphosis development in *B. dorsalis*.

In this study, we identified *B. dorsalis* miRNAs at three stages of metamorphosis development, WS, LWS and WPS, using deep sequencing. We detected 249 miRNAs, including 184 known miRNAs and 65 novel miRNAs. This result is comparable to the findings of Tariq et al. (2016), who reported 172 known and 78 novel miRNAs from the testes of fully mature, immature, and middle-aged adult flies of *B. dorsalis*. Our data shows a small difference in novel miRNAs when compared with the report by Peng et al. (2016), in which data analysis revealed 183 known and 120 novel miRNAs from the whole body of females and males, the ovaries and the testes of *B. dorsalis*. Our data has detected significantly more miRNAs than the early report by Huang et al. (2014), in which only 123 known and 60 novel miRNAs were identified among different developmental stages (eggs, larvae, pupae, and adults) in *B. dorsalis*. We were able to detect increased numbers of known and novel miRNAs in our study compared to Huang et al. (2014) because of the availability of the whole genome sequence (WGS) of *B. dorsalis*, and compared with Peng et al. (2016) because of the upgraded miRBASE database.

When pairwise comparison of the DEMsbetween the three developmental stages (WS vs. LWS and LWS vs. WPS) was carried out, 19 miRNAs were found which eight miRNAs showed relative high expression levels. Highly expressed DEMs are of interest, for example, Reynolds et al. (2017) selected high abundance DEMs from the transcriptome data and other conditional miRNAs to study further. In our study, eight highly expressed levels DEMs, miR-2-3p, miR-282-5p, let-7a-3p, miR-981, miR-100, Bdo-novel-mir-24, Bdo-novel-mir-44 and Bdo-novel-mir-55, were detected during pupariation of *B. dorsalis*, which indicates that they may have regulatory functions. Previous studies have discussed these known DEMs in other insects. The miR-2 family targets *awd* and *fng* to regulate wing morphogenesis in the holometabolous species, *B. mori* (Ling et al., 2015) and regulates insect metamorphosis by controlling the JH signaling pathway gene *Kr-h1* in the hemimetabolous species, *Blattella germanica* (Lozano et al., 2015). miR-282 regulates the nervous system-specific adenylate cyclase (*rutabaga*) to control the viability, longevity, and egg production of *Drosophila melanogaster* (Vilmos et al., 2013), and has been shown by Solexa sequencing to be differentially expressed between foraging and dancing honey bees, *Apis mellifera*, and to target the genes associated with kinases, neural function, synaptotagmin, and energy (Li et al., 2012). miR-958 inhibits Toll signaling and Drosomycin expression by direct targeting of *Toll* and *Dif* in *D. melanogaster* (Li S. et al., 2017). miR-100 regulates wing morphogenesis and may be related to the *Broad complex* gene, an early 20E response gene in the ecdysteroid cascade, in *B. germanica* (Rubio and Belles, 2013). Therefore, the eight DEMs, combined with their predicted target mRNAs, may be associated with the regulation of metamorphosis.

Using the miRanda and RNAhybrid software packages, the target genes of the DEMs detected during metamorphosis development were predicted. There were 12 and 4 DEMs with predicted target genes in WS vs. LWS and LWS vs. WPS, respectively. Although some DEMs were not predicted to have mRNA target genes, this does not mean that they have no regulatory role, and they may work through other regulatory factors (such as long non-coding RNAs and circular RNAs) to influence pupariation. Braconi et al. (2011) showed that miRNA-29 can regulate expression of the long non-coding RNA gene *MEG3*, and Ebbesen et al. (2016) observed that circular RNAs can function as miRNA sponges acting as protein decoys, or transcriptional regulators of mRNA. When the miRNAs were differentially expressed during metamorphosis development, their predicted mRNA target genes were also differentially expressed, indicating that the miRNAs may participate in this progress, especially when the DEMs and their predicted target genes were observed in a miRNA–mRNA negative regulation pattern, as this is the typical pattern seen between miRNAs and their target genes. For instance, miR-71 and miR-263 negatively regulate their target genes *chitin synthase 1* and *chitinase 10*, respectively, to control locust molting in *L. migratoria* (Yang et al., 2016); miRNA-252-5p negatively controls the target gene *Abelson interacting protein* to decrease the protein levels of cyclins A and B, controlling the cell cycle in *Drosophila* (Lim et al., 2018). Similarly, miR-9a directly negatively regulates the target gene *adenylyl cyclase 2* in *L. migratoria* (Guo X. et al., 2018). There were no DEMs and corresponding DEGs in the comparison of LWS vs. WPS once this negative regulation pattern was considered. This is probably because the two stages are so close that there are few differences between LWS and WPS, and there were few differentially expressed genes in the RNA-seq analysis for the comparison of the two stages (Chen E. H. et al., 2018). The subsequent qPCR results also showed that there were no significant differences between the verified miRNAs and mRNAs at the two stages.

To further understand the potential function of the DEMs during pupariation, their predicted target genes were annotated. A GO classification map was generated by gene enrichment analysis of the DEMs and their predicted target DEGs. This classification map could be useful for further study into how the DEMs are related to metamorphosis development. Considering of previously reported genes related to insect pupariation, the DEGs were expected to be mapped to the genes involved in 20E, JH, chitin pathways, cuticular proteins and etc. The RNAi-mediated knockdown of ecdysone synthesis pathway genes (*CYP306A1* and *CYP314A1*) caused lethality and slowed down ecdysis during *Leptinotarsa decemlineata* nymphal stages (Kong et al., 2014; Wan et al., 2014). Silencing of both *E74* isoforms (*E74A* and *E74B*) caused failure of ecdysis, and most beetles after *LdE74* RNAi remained as prepupae while a few larvae finally became deformed pupae, with shortened antennae and legs in *L. decemlineata* (Xu et al., 2018). RNA interference-mediated knockdown of *juvenile hormone acid O-methyltransferase* (*JHAMT*) (Minakuchi et al., 2008) or *Kr-h1* (Minakuchi et al., 2009) in the *Tribolium castaneum* larval stage both resulted in precocious larval–pupal metamorphosis. In *Spodoptera exigua*, injection of *chitinase* dsRNA at the larval–pupal stage caused lethal phenotype with visible pupal cuticle under the old larval cuticle, indicating that *chitinase* participated in the degradation of old cuticle during insect pupariation (Zhang et al., 2012). When silencing *chitin synthase 1* in third-instar larvae of *B. dorsalis*, the larvae were trapped their old cuticle and died without pupation (Yang et al., 2013). In a recently published paper about *B. dorsalis*, some key DEGs were annotated in RNA-seq analysis during pupariation, for example, 20E biosynthesis (*CYP314a1*) and signaling pathway genes (*E74*), JH synthesis (*farnesoic acid O-methyl transferase*), chitinases (*chitinase 3* and *5*), and serine proteases (Chen E. H. et al., 2018). Unexpectedly, the predicted target DEGs in the current study were largely unsuccessfully mapped to those genes above. One of the reasons might be that some genes related to insect pupariation are not regulated by miRNAs. A previous study pointed out that only about 30% of human genes are regulated by microRNAs (Lewis et al., 2005). Namely, the majority of organism genes are not regulated by microRNAs. In addition, other crucial genes were not significant different (|log2Ratio| < 1) in expression during pupariation, for example, 20E biosynthesis (*CYP315a1*), JH synthesis (*3-Hydroxy-3-methylglutaryl-CoA reductase*) and signaling pathway genes (*Met* and *Kr-h1*), chitin synthases (*glucose-6-phosphate isomerase*) (Chen E. H. et al., 2018). Furthermore, the miRNAs regulation is temporal during growth and development stages (Belles, 2017). Nevertheless, serine proteases and chitinase reported in this process were annotated in predicted target genes of DEMs. Among the Nr annotation of the predicted DEGs of the five highly expressed DEMs, two of the genes, *spsX1* (Paskewitz et al., 1999) and *sp1/2*, were related to serine proteases, which have been suggested to be involved in integument remodeling during metamorphosis (Gu et al., 2013; Liu H. W. et al., 2017). However, these two genes showed contrasting changes in expression levels (*spsX1* was up-regulated and sp1/2 was down-regulated), indicating that the serine proteases may be regulated by different miRNAs. During metamorphosis, high expression levels of serine proteases in the epidermis may be required to break down the cuticle proteins and release the chitin so that it can be degraded (Gu et al., 2013). Chitinase 3, a member of the chitinase family, is responsible for chitin degradation. During pupariation, the chitin needs to be degraded and replaced by newly synthesized chitin. Here, we found that Bdo-novel-mir-55 probably targets chitinase 3 to control *B. dorsalis* metamorphosis. Chitinase can be regulated by miRNAs and the regulation has been confirmed in previous studies. For example, miR-263 mediates the gene encoding *chitinase 10* in *L. migratoria* (Yang et al., 2016), and miR-24 targets *chitinase* to regulate the molting process of *Helicoverpa armigera* (Agrawal et al., 2013). Furthermore, the genes *protein skeletor* (Sengupta et al., 2016) and *lap* (Rabossi et al., 2000) have been reported to be involved in metamorphosis. As the majority of these predicted target genes are related to the process of metamorphosis development, in combination with the qPCR results, we hypothesize that miR-981 may target *pdpc*, Bdo-novel-mir-55 potentially regulates *spsX1, psB/C* and *chit3*, let-7a-3p possibly targets *lap*, and Bdo-novel-mir-24 may regulate *ipc* and *sp1/2* to govern the metamorphosis of *B. dorsalis*. Undoubtedly, the relationship of regulation between DEMs and predicted target genes deserves validating further. The miRNAs-mediated mechanism underlying pupariation is complex and the systematic interpretation deserves further work (i.e., definition of crucial development node of pupariation).

## Conclusion

In this study, HiSeq combined with RNA-seq analysis was performed to elucidate the molecular regulatory mechanisms of small RNAs in *B. dorsalis* during pupariation. One hundred twenty-eight miRNAs were differentially expressed, which indicates that these DEMs may play important regulatory roles in the process of pupariation. The numbers of up-regulated and down-regulated DEMs were approximately even. There were eight miRNAs showing relative high expression levels (>50 TPM), of which five DEMs had predicted DEGs according to the expected miRNA–mRNA negative regulation pattern using the DESeq R algorithm, and four pairs of DEMs and their predicted DEGs were confirmed by qPCR. Of these four miRNAs, two miRNAs were down-regulated, with miR-981 potentially targeting *pdpc* and Bdo-novel-mir-55 potentially regulating *spsX1, psB/C* and *chit3*, and the other two miRNAs were up-regulated, with let-7a-3p possibly controlling *lap* and Bdo-novel-mir-24 perhaps managing *ipc* and *sp1/2*. Overall, our study provides basic miRNA expression data that will aid the exploration of the regulatory mechanisms of metamorphosis by miRNAs in *B. dorsalis*. Verification of the regulatory relationships between these miRNAs and their target genes is needed to further uncover the possible roles of these miRNAs in *B. dorsalis* pupariation.

## Author Contributions

QZ, WD, E-HC, J-ZN, and GS contributed conception and design of the study. QZ and DP organized the database. QZ and J-JW performed the statistical analysis. QZ wrote the first draft of the manuscript. QZ, WD, J-ZN, J-JW, and GS wrote sections of the manuscript. All authors contributed to manuscript revision, read and approved the submitted version.

## Conflict of Interest Statement

The authors declare that the research was conducted in the absence of any commercial or financial relationships that could be construed as a potential conflict of interest.

## Footnotes

1 http://www.mirbase.org/

2 http://www.ncbi.nlm.nih.gov/Structure/bwrpsb/bwrpsb.cgi

3 http://www.ncbi.nlm.nih.gov/tools/primer-blast/

## Supplementary Material

FIGURE S1 GO classification analysis for predicted differentially expressed genes (DEGs) of differentially expressed miRNAs (DEMs) in WS vs. LWS. (All secondary level was under the backgrounds of the predicted DEGs and the predicted target genes of DEMs).

FIGURE S2 COG classification analysis for predicted differentially expressed genes (DEGs) of differentially expressed miRNAs (DEMs) in WS vs. LWS.

FIGURE S3 KEGG classification analysis for predicted differentially expressed genes (DEGs) of differentially expressed miRNAs (DEMs) in WS vs. LWS.

TABLE S1 Primers of miRNA and mRNA used in our study.

TABLE S2 Overview of sRNA sequencing data.

TABLE S3 Mapping information of unannotated reads with reference genome.

TABLE S4 List of total identification, respective and overlapped known and novel miRNAs in three developmental stages of *Bactrocera dorsalis*.

TABLE S5 List of differentially expressed miRNAs in WS vs. LWS and LWS vs. WPS of *Bactrocera dorsalis*.

TABLE S6 List of 19 differentially expressed miRNAs in the pupariation of *Bactrocera dorsalis*.

TABLE S7 List of annotation of differentially expressed miRNAs in WS vs. LWS and LWS vs. WPS of *Bactrocera dorsalis*.

TABLE S8 List of differentially expressed miRNAs with changed predicted target mRNAs in WS vs. LWS of *Bactrocera dorsalis*.

TABLE S9 List of miRNAs and predicted target mRNAs coinciding with the miRNA–mRNA negative regulation pattern in WS vs. LWS of *Bactrocera dorsalis*.
