# Supplementary material for: Genome-Wide Analysis of MicroRNAs in Relation to Pupariation in Oriental Fruit Fly
Source: Front Physiol. 2019 Mar 22;10:301. doi: 10.3389/fphys.2019.00301 (PMC6439999; doi:10.3389/fphys.2019.00301)
Supplement: FIGURE S1 — GO classification analysis for predicted differentially expressed genes (DEGs) of differentially expressed miRNAs (DEMs) in WS vs. LWS. (All secondary level was under the backgrounds of the predicted DEGs and the predicted target genes of DEMs). [file Image_1.pdf]

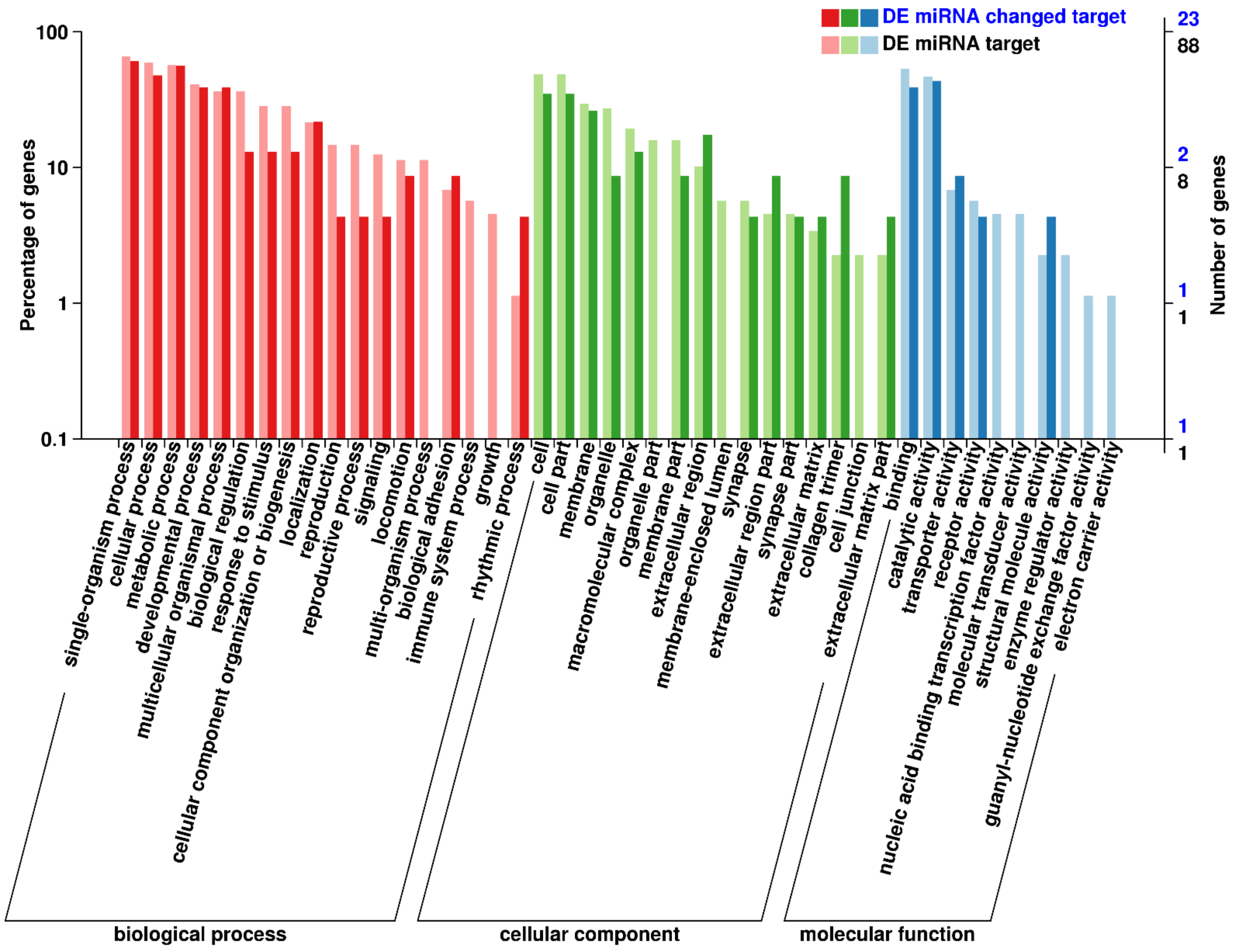

Fig S1. GO classification analysis for predicted differentially expressed genes (DEGs) of differentially expressed miRNAs (DEMs) in WS vs. LWS. (all secondary level was under the backgrounds of the predicted DEGs and the predicted target genes of DEMs)
