# Supplementary material for: Genome-Wide Analysis of MicroRNAs in Relation to Pupariation in Oriental Fruit Fly
Source: Front Physiol. 2019 Mar 22;10:301. doi: 10.3389/fphys.2019.00301 (PMC6439999; doi:10.3389/fphys.2019.00301)
Supplement: FIGURE S2 — COG classification analysis for predicted differentially expressed genes (DEGs) of differentially expressed miRNAs (DEMs) in WS vs. LWS. [file Image_2.pdf]

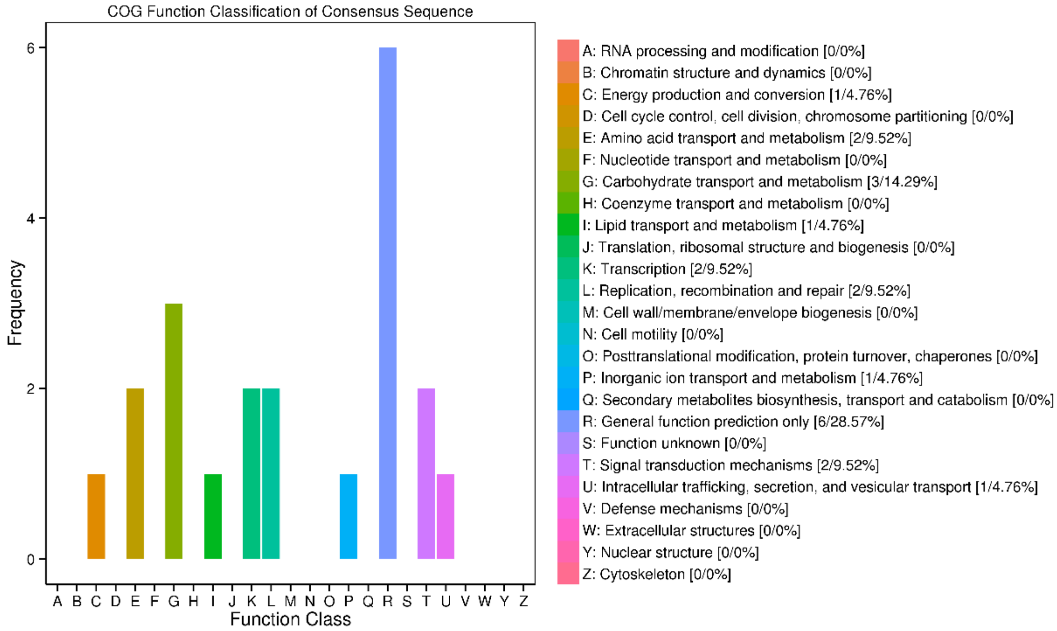

Figure S2. COG classification analysis for predicted differentially expressed genes (DEGs) of differentially expressed miRNAs (DEMs) in WS vs. LWS.
