# Supplementary material for: Genome-Wide Analysis of MicroRNAs in Relation to Pupariation in Oriental Fruit Fly
Source: Front Physiol. 2019 Mar 22;10:301. doi: 10.3389/fphys.2019.00301 (PMC6439999; doi:10.3389/fphys.2019.00301)
Supplement: TABLE S2 — Overview of sRNA sequencing data. [file Table_2.docx]

| **Samples** | **Raw_reads** | **Low_quality** | **Containing'N'reads** | **Length<18** | **Length>30** | **Clean_reads** | **Q30(%)** |
| --- | --- | --- | --- | --- | --- | --- | --- |
| WS-1 | 19954889 | 0 | 426 | 1190525 | 1429599 | 17334339 | 98.97 |
| WS-2 | 28621768 | 0 | 702 | 1237804 | 3202199 | 24181063 | 99.02 |
| WS-3 | 28834258 | 0 | 651 | 1003931 | 5545475 | 22284201 | 99.03 |
| LWS-1 | 39539757 | 0 | 895 | 2118321 | 4260439 | 33160102 | 99.01 |
| LWS-2 | 17837137 | 0 | 425 | 5409529 | 535095 | 11892088 | 98.94 |
| LWS-3 | 21675562 | 0 | 465 | 409816 | 5616590 | 15648691 | 98.98 |
| WPS-1 | 23765595 | 0 | 521 | 556458 | 6127076 | 17081540 | 98.97 |
| WPS-2 | 25451738 | 0 | 585 | 1301339 | 3567732 | 20582082 | 99.05 |
| WPS-3 | 17660363 | 0 | 765 | 5201463 | 590764 | 11867371 | 98.87 |

Table S2. Overview of sRNA sequencing data.
