# Supplementary material for: Genome-Wide Analysis of MicroRNAs in Relation to Pupariation in Oriental Fruit Fly
Source: Front Physiol. 2019 Mar 22;10:301. doi: 10.3389/fphys.2019.00301 (PMC6439999; doi:10.3389/fphys.2019.00301)
Supplement: TABLE S3 — Mapping information of unannotated reads with reference genome. [file Table_3.docx]

Table S3 Mapping information of unannotated reads with reference genome.

| Samples | Total_reads | Mapped_reads | Mapped_reads(+) | Mapped_reads(-) | Mapped rate(%) |
| --- | --- | --- | --- | --- | --- |
| WS-1 | 11537570 | 3924773 | 2017993 | 1906780 | 34.02 |
| WS-2 | 15424364 | 5477943 | 2584281 | 2893662 | 35.51 |
| WS-3 | 15020184 | 5124970 | 2488046 | 2636924 | 34.12 |
| LWS-1 | 18733121 | 4978780 | 2541298 | 2437482 | 26.58 |
| LWS-2 | 6691682 | 2076970 | 1089962 | 987008 | 31.04 |
| LWS-3 | 8548301 | 1841624 | 983977 | 857647 | 21.54 |
| WPS-1 | 8934016 | 2521417 | 1354865 | 1166552 | 28.22 |
| WPS-2 | 11442076 | 3815585 | 1882550 | 1933035 | 33.35 |
| WPS-3 | 6193156 | 2042870 | 1054252 | 988618 | 32.99 |

Total_Reads: unannotated reads; Mapped_Reads: reads mapped successfully into the reference genome; Mapped_reads(+): reads mapped successfully into the reference genome (plus strand); Mapped_reads(-): reads mapped successfully into the reference genome (minus strand); Mapped rate(%): The ratio of mapped_reads to total_reads
